# Assessing the Effect of Different Teaching Methods on Left Atrial‐to‐Aortic Ratio Image Acquisition and Image Interpretation

**DOI:** 10.1111/vec.70104

**Published:** 2026-04-15

**Authors:** Kathrin Siedenburg, Karen Humm, Simon David Cook, Joshua Hannabuss, Laura Pearl Cole

**Affiliations:** ^1^ Department of Clinical Science and Services The Royal Veterinary College Hatfield UK; ^2^ University of Veterinary Medicine Hannover Hannover Germany

**Keywords:** cardiac ultrasound, teaching methods, veterinary education

## Abstract

**Objective:**

To determine the effect of three different teaching methods on left atrial‐to‐aortic ratio (LA:Ao) image acquisition and interpretation, student confidence, and knowledge retention.

**Design:**

Prospective, educational study.

**Setting:**

University veterinary teaching hospital.

**Study Subjects:**

Thirty‐six fourth‐year preclinical veterinary students and three dogs without left atrial enlargement.

**Interventions:**

Students were randomized into three groups (A, B, and C). All students watched a teaching video on acquiring and interpreting the LA:Ao view. Groups B and C received in‐person teaching. Group C received instructor‐led, hands‐on training. Students completed a confidence survey before the teaching intervention (T0), after the teaching intervention (T1), and 7–8 months later (T2). LA:Ao image acquisition (live dog) and interpretation (from stills) were assessed at T1 and T2.

**Measurements and Main Results:**

Student confidence and teaching method preferences were assessed using a 5‐point Likert‐type scale at T0, T1, and T2. LA:Ao image acquisition and interpretation were assessed at T1 and T2. Student confidence in image interpretation improved in all groups; only Group C had improved confidence in operating the ultrasound machine. At T1, interpretable LA:Ao views were acquired by two of 10, four of 12, and seven of 12 students from Groups A, B, and C, respectively, and at T2 by two of five, three of six, and four of seven students from Groups A, B, and C, respectively. The median image interpretation test score at T1 was 5/5 (interquartile range [IQR]: 5), 4/5 (IQR: 4–5), and 4/5 (IQR: 3–5) for Groups A, B, and C, respectively. At T2, the median image interpretation scores were 3/5 (IQR: 2–4), 3.5/5 (IQR: 3–4), and 4/5 (IQR: 3–5) for Groups A, B, and C, respectively.

**Conclusions:**

LA:Ao image acquisition was achieved in more students following instructor‐led, hands‐on teaching; however, this difference was not significant, possibly due to small group sizes. Video training alone appeared to be adequate for LA:Ao image interpretation.

**Abbreviations**
ECC: emergency and critical care
FCU: focused cardiac ultrasound
IQR: interquartile range
LA:Ao: left atrial‐to‐aortic ratio
POCUS: point‐of‐care ultrasound

## 1 Introduction

Point‐of‐care ultrasound (POCUS) has become integral in the veterinary emergency and critical care (ECC) setting for patient assessment. Focused cardiac ultrasound (FCU) is a quick examination involving targeted ultrasonographic views to identify gross abnormalities in cardiac chamber size or systolic function [1]. Evaluating the left atrial‐to‐aortic ratio (LA:Ao) is one aspect of FCU assessment to detect left atrial enlargement [2].

The international guidelines for teaching FCU in human medicine strongly recommend integrating FCU into the medical school curricula [3]. Hands‐on practical POCUS training in people has consistently been shown to be superior to didactic training for image acquisition but not consistently for image interpretation. Instructor‐led, hands‐on training, through dedicated supervised sessions, has been shown to improve image acquisition and knowledge of POCUS in human training and is often preferred by students [4, 5]. No difference in image acquisition and interpretation has been demonstrated between web‐based and in‐person didactic training [6].

In people, hands‐on training for teaching of FCU image acquisition and interpretation is strongly recommended [3]. The application of FCU has also been found to be beneficial in diagnosing cardiac disease in small animals [7, 8, 9, 10]. Hands‐on FCU training in nonspecialized veterinarians has been shown to improve proficiency in image acquisition and image interpretation, as well as increasing user confidence [3, 6, 7, 11]. Few studies have evaluated POCUS training methods in veterinary students. One study comparing the effect of live animal training to online video instruction on students’ proficiency in abdominal focused assessment with sonography for trauma examinations showed that an instructional video alone is likely appropriate for developing basic ultrasound skills, but additional training may be necessary to acquire more difficult sonography skills [12]. A study specifically looking at FCU training showed that student confidence in measuring left ventricular and LA:Ao dimensions increased when in‐person didactic training was combined with self‐directed, hands‐on training [11]. Another veterinary institution described the logistic process involved in developing a basic ultrasound curriculum for sonographic assessment of the canine abdomen, including simulation training and live‐dog scanning, and evaluated the success of its implementation [13].

While the effectiveness of student teaching is paramount, the method of training must also be accessible, cost‐efficient, and time resourceful when large student cohorts are involved. Instructor‐led, hands‐on training with live dogs and in‐person skills demonstration are both costly and time‐consuming. Web‐based training increases accessibility and is more cost effective and potentially more feasible for training large numbers of students. If a teaching method that is more accessible, affordable, and scalable can be developed while also maintaining the same effectiveness as resource‐intensive methods, POCUS training could be more readily integrated into the veterinary curricula.

The aims of the current study were to evaluate three different methods for teaching sonographic LA:Ao assessment to veterinary students: web‐based training alone, web‐based training with in‐person teaching, and web‐based training with in‐person teaching and instructor‐led, hands‐on training. The objective was to compare the effect of the different teaching methods on LA:Ao image acquisition, image interpretation, student confidence, and knowledge retention. We hypothesized that web‐based training alone would be inferior to the addition of in‐person teaching when assessed by any of the measured outcomes (i.e., image acquisition, image interpretation, knowledge retention, and student confidence). Additionally, we hypothesized that instructor‐led, hands‐on training would result in improved image acquisition, knowledge retention, and student confidence but would not improve image interpretation.

## 2 Materials and Methods

**FIGURE 1:**
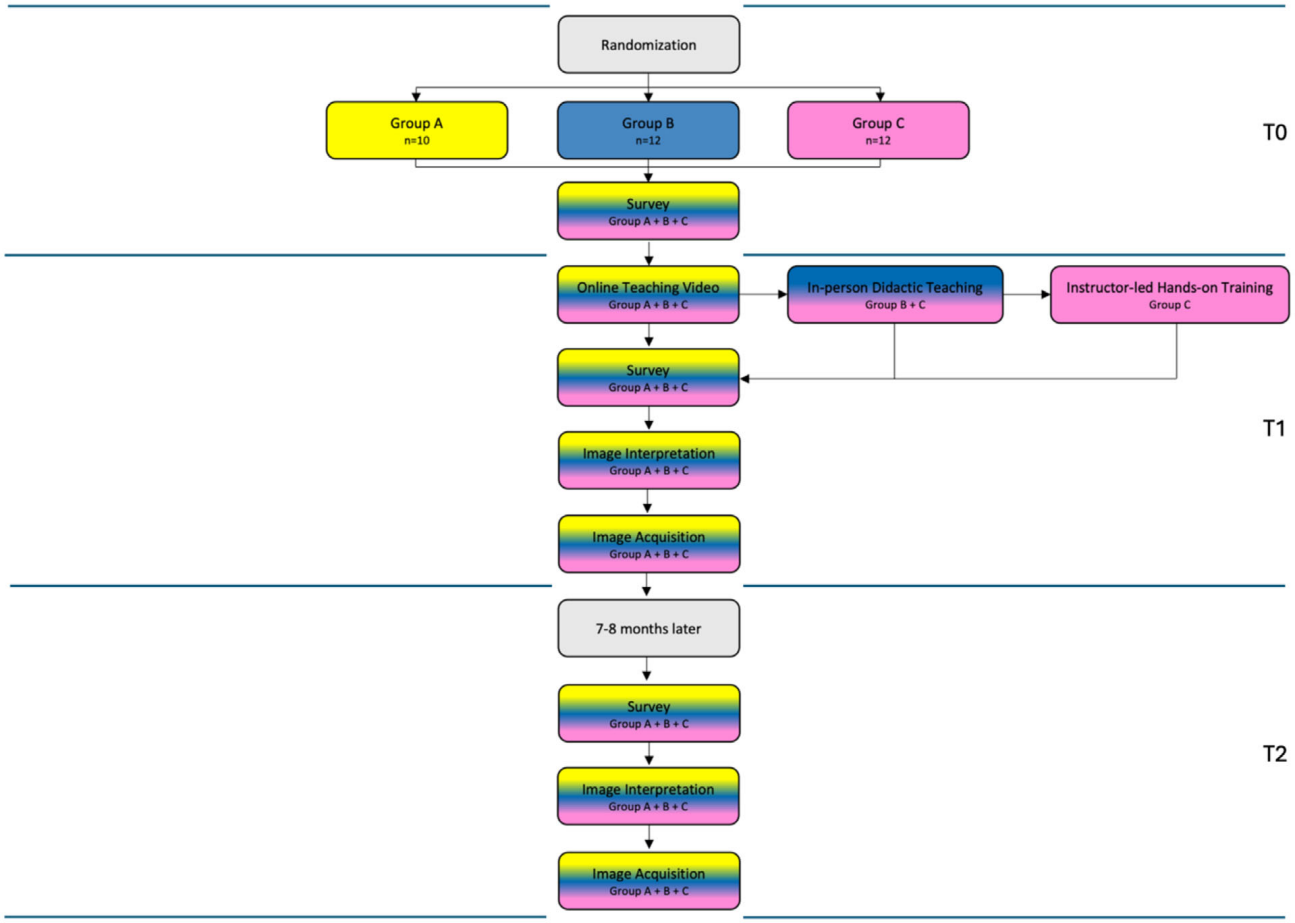
Flow chart of the prospective design of a study in preclinical veterinary students who watched a teaching video on acquiring and interpreting the LA:Ao view (Groups A, B, and C); who then also received in‐person teaching (Groups B and C); and who then also received instructor‐led, hands‐on training (Group C).

This prospective randomized study was approved by the Clinical Research Ethical Review Board (URN 2022 2139‐2) and the Social Sciences Research Ethical Review Board (SR2022‐0003) at The Royal Veterinary College, Hatfield, UK. The timeline of the study is shown in Figure 1.

Two hundred ninety‐six fourth‐year veterinary students (enrolled in a 5‐year undergraduate veterinary medicine degree at a single academic institution) who had not yet undergone clinical rotations were contacted via email and offered voluntary participation in the training course. Thirty‐six participation spots were offered.

Students who had already participated in clinical rotations were excluded. Preexisting experience with ultrasonography was not an exclusion criterion. Participant consent was obtained before study commencement. Randomization software^1^ was used to assign participants into one of three teaching groups using block randomization (A: web‐based teaching video; B: web‐based teaching video and in‐person teaching session; C: web‐based teaching video and in‐person teaching session and instructor‐led, hands‐on training). The study was extracurricular, and no compensation, either monetary or as academic credit, was provided for participation.

A web‐based survey was conducted on the first study day in all groups before any teaching intervention (T0) (Supporting Information S1). In this survey, students’ ultrasonographic experience (in quality and quantity), confidence in performing ultrasound, and teaching method preference were surveyed. For evaluation of student confidence and teaching method preference, a 5‐point Likert‐type scale (ranging from 1 = *strongly disagree* to 5 = *strongly agree*) was used (e.g., survey statement, “I feel confident in operating an ultrasound machine.”). An overall student confidence score was derived by summing the Likert‐type scale scores from four domains (confidence in ultrasound machine operation, anatomic landmark localization, left atrial enlargement detection, and correct LA:Ao image interpretation).

After the survey, all three groups watched an 11‐min online teaching video on ultrasound machine operation and acquisition and interpretation of the LA:Ao view in the dog (Supporting Information S2). Groups B and C then received an in‐person teaching session including questions and answers, with 12 students per teaching session. Using a live dog with no underlying cardiac abnormalities, a second‐year ECC resident demonstrated a 15‐min practical stepwise approach on how to acquire an interpretable LA:Ao image.

After the in‐person didactic teaching session, Group C participated in a 1‐h instructor‐led, hands‐on practical training session conducted by a second‐year ECC resident and a board‐certified specialist in ECC. There were six students in each of two groups, with all students having 10 min to practice LA:Ao image acquisition on a live dog under one‐on‐one supervision by either the resident or the diplomate. During this time, instructors provided individual teaching, including demonstration of ultrasound probe movement and hands‐on adjustment for correct LA:Ao image acquisition and live feedback. During this teaching session, students were able to observe one another, ask questions, and request direct individual feedback on their performance.

A second web‐based survey was conducted immediately after students had completed their assigned teaching intervention(s) (T1). Student confidence in ultrasound and teaching method preference were re‐assessed. Additionally, students’ satisfaction with their assigned teaching intervention was surveyed using a 5‐point Likert scale and open questions (Supporting Information S3). LA:Ao image interpretation skills were then evaluated with a five‐question web‐based test (Supporting Information S4).

Finally, LA:Ao image acquisition skills were assessed. Participants were required to obtain one image of the LA:Ao view on a live dog within 5 min. If they obtained multiple images within the given time, they were asked to choose the one that they thought would be best for evaluation of image acquisition accuracy and quality. To assess their ability to critique the image quality for suitability of interpretation, students were asked to classify their chosen image as (1) interpretable or (2) noninterpretable. Each image was evaluated by a third‐year cardiology resident, blinded to the assigned teaching group. Images were assessed using the following three anatomic criteria: aortic symmetry and shape, presence and shape of the left auricle, and presence and shape of the pulmonary artery. Images were classified as (1) interpretable or (2) noninterpretable.

All students were invited back 7–8 months later to complete re‐evaluation (T2) and were asked to complete a survey assessing their experience and confidence in ultrasonographic performance alongside their teaching method preference (Supporting Information S5). After completion of the survey, image interpretation and image acquisition were re‐evaluated.

### 2.1 Ultrasonographic Device

Throughout the study, two pocket‐sized, handheld ultrasound probes operating at 1–10 MHz^2^, each connected to an electronical tablet^3^ that allowed visualization of the ultrasound image via the application provided with the probes, were used as sonographic devices.

### 2.2 Instructors and Their Content Expertise and Education Background

Two instructors, a second‐year ECC resident and a board‐certified ECC specialist and fellow of the higher education academy, both having at least 3 years of FCU experience in an undergraduate and postgraduate teaching hospital, provided the course material and acted as instructors for the instructor‐led, hands‐on training. Only the resident provided the in‐person didactic teaching.

### 2.3 Animals

The staff at the academic institution were contacted via email and asked to voluntarily allow their privately owned dogs to participate in the study as live sonographic models. Inclusion criteria were medium‐ to large‐sized dogs with a calm demeanor and no underlying left atrial enlargement. A FCU was performed by a second‐year ECC resident to rule out underlying left atrial enlargement. Owner consent was obtained before enrollment. The dogs’ fur was not clipped for this study, but local application of alcohol and ultrasound gel was used to improve image quality.

### 2.4 Data Analysis

Data analysis was performed using a commercially available statistical software^4^. A Shapiro–Wilk test was used to assess for normality of data distribution. Data were expressed as median (interquartile range [IQR]). A Fisher exact test was used to compare preexisting ultrasound experience between groups at T0 and image acquisition between groups at T1. A Mann–Whitney *U*‐test was used to compare confidence scores of students who successfully acquired an interpretable LA:Ao image at T1 and students who did not. A Kruskal–Wallis test was used to compare image interpretation scores between groups at T1 as well as teaching method preference at T0 and T1. A Wilcoxon signed‐rank test followed by a Dunn–Bonferroni post hoc analysis was performed for pairwise comparison of Likert‐type scale student confidence scores and teaching method preference scores for comparisons over time (T0 to T1 only). Statistical significance was set at *p* < 0.05. Due to small group sizes, data acquired at T2 were only reported descriptively. Qualitative data analysis involved inductive thematic analysis of open‐ended survey questions. Analyzing and coding of qualitative data were performed by the primary author and confirmed by a coauthor.

## 3 Results

Three privately owned dogs of different breeds (Miniature Australian Shepherd, Lurcher, German Shorthaired Pointer), aged between 5 and 10 years, with a body weight ≥13 kg but ≤30 kg were enrolled in the study.

Thirty‐six students volunteered for participation in the study. All 36 were enrolled and randomized into three groups, resulting in 12 students per group. Two students from teaching Group A were excluded because of lack of participation, for a total of 34 students participating in the study (Group A: 10, Group B: 12, Group C: 12). One student from Group B did not complete the survey after the teaching intervention and was therefore not included in assessment of these data, but all other data relating to the student were included. Eighteen of 34 students (53%) who had completed T0 and T1 of the study participated in T2; however, one student from Group A did not complete the survey or the image interpretation test and was therefore not included in assessment of these data, but image acquisition data relating to the student were included.

At T0, most students (26/34 [76%]) had previously operated an ultrasound machine (8/10, 10/12, and 8/12 participants in Groups A, B, and C, respectively). Thirteen of 34 (38%) students stated they had previously performed cardiac ultrasound (3/10, 6/12, and 4/12 in Groups A, B, and C, respectively). No significant difference was seen in preexisting experience between groups. At T2, 13 of 17 students (76% [3/4, 4/6, and 6/7 in Groups A, B, and C, respectively]) had operated an ultrasound machine since T1. When specifically asked about cardiac ultrasound, eight of 17 students (47% [1/4, 2/6, and 5/7 in Groups A, B, and C, respectively]) had previously performed cardiac ultrasound.

### 3.1 Image Acquisition

**FIGURE 2:**
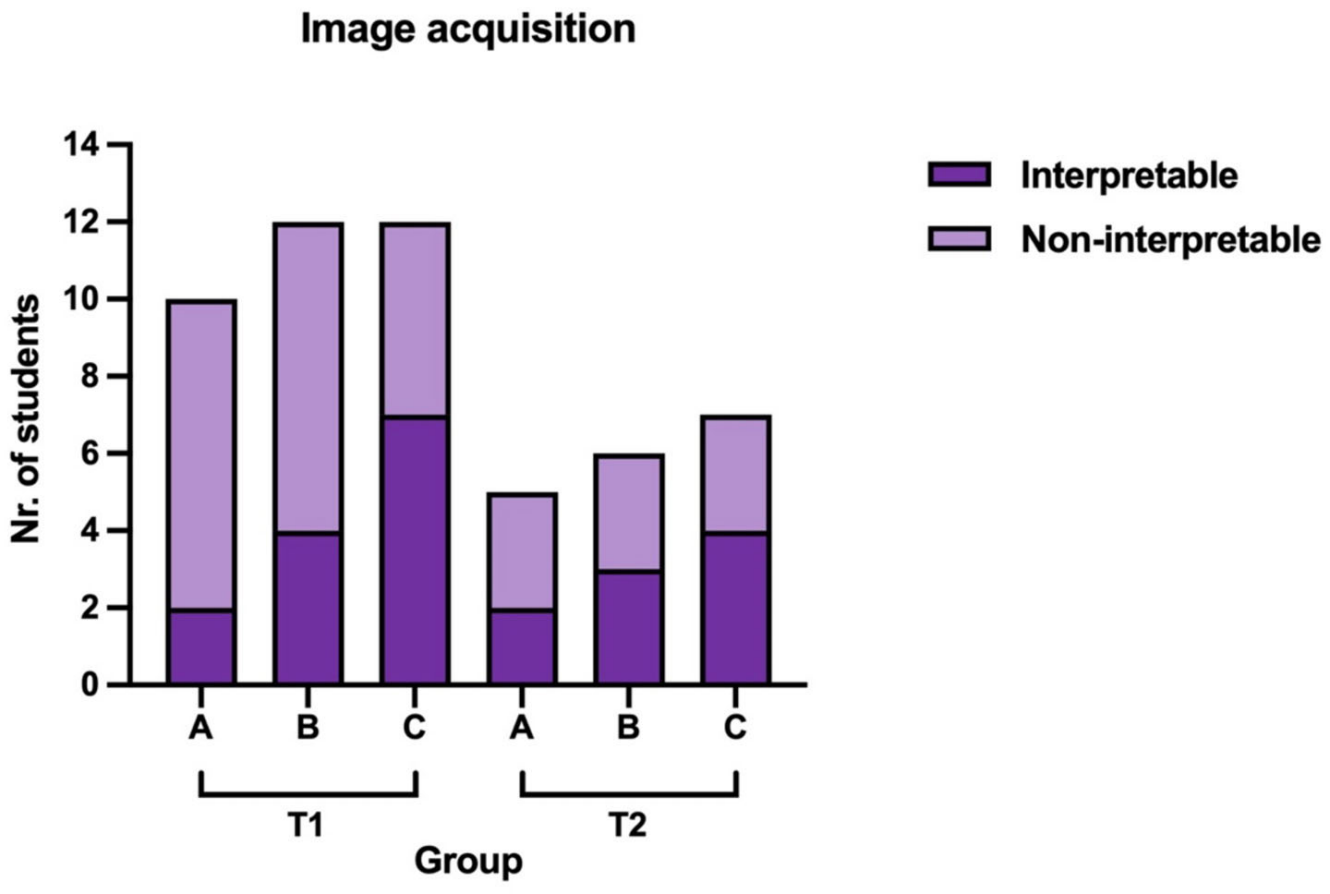
Number of preclinical veterinary students who acquired an interpretable versus a noninterpretable LA:Ao image at T1 (immediately after training) and T2 (7–8 months later) in Groups A, B, and C. All students (Groups A, B, and C) watched a teaching video on acquiring and interpreting the LA:Ao view; Groups B and C then also received in‐person teaching; and Group C then also received instructor‐led, hands‐on training. LA:Ao, left atrial‐to‐aortic ratio.

**FIGURE 3:**
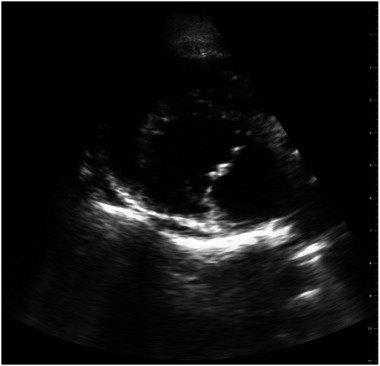
Ultrasonographic LA:Ao image from a live dog, acquired during the image acquisition assessment, classified as noninterpretable by the cardiologist and interpretable by the student who acquired the image. LA:Ao, left atrial‐to‐aortic ratio.

**FIGURE 4:**
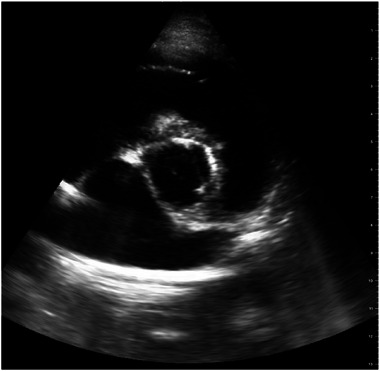
Ultrasonographic LA:Ao image from a live dog, acquired during the image acquisition assessment, classified as interpretable by the cardiologist and noninterpretable by the student who acquired the image. LA:Ao, left atrial‐to‐aortic ratio.

At T1, interpretable LA:Ao views were acquired by two of 10, four of 12, and seven of 12 students of Groups A, B, and C, respectively (Figure 2). Successful image acquisition did not differ among groups (*p* = 0.204). When students with previous cardiac ultrasound experience were excluded, one of seven, two of six, and three of eight students of Groups A, B, and C, respectively, successfully acquired an interpretable LA:Ao image. The proportion of students with successful image acquisition did not differ between those with and without preexisting ultrasound experience (*p* = 0.298), nor did it differ in those with and without previous specific cardiac ultrasound experience (*p* = 0.167). Twenty of 32 students correctly assessed the interpretability of their acquired LA:Ao image (interpretable vs. noninterpretable) when compared with the assessment of the cardiology resident. Seven of 20 students misclassified their image as being interpretable (Figure 3), and five of 20 students misclassified their image as being noninterpretable (Figure 4). Two students declined to self‐reflect on the interpretability of their LA:Ao image. At T2, interpretable LA:Ao views were acquired by two of five, three of six, and four of seven students in Groups A, B, and C, respectively. Thirteen of 17 students correctly assessed the interpretability of their acquired LA:Ao image (interpretable vs. noninterpretable) when compared with the assessment of the cardiology resident. Three of four students misclassified their image as being interpretable, and one student misclassified their image as being noninterpretable. One student declined to self‐reflect on the interpretability of their LA:Ao image.

### 3.2 Image Interpretation

At T1, the median image interpretation test scores (out of total 5) were 5 (IQR: 5), 4 (IQR: 4–5), and 4 (IQR 3–5) for Groups A, B, and C, respectively. No difference among the groups was detected (*p* = 0.073). The median image interpretation scores at T2 were 3 (IQR: 2–4), 3.5 (IQR: 3–4), and 4 (IQR: 3–5) for Groups A, B, and C respectively.

### 3.3 Student Confidence

**FIGURE 5:**
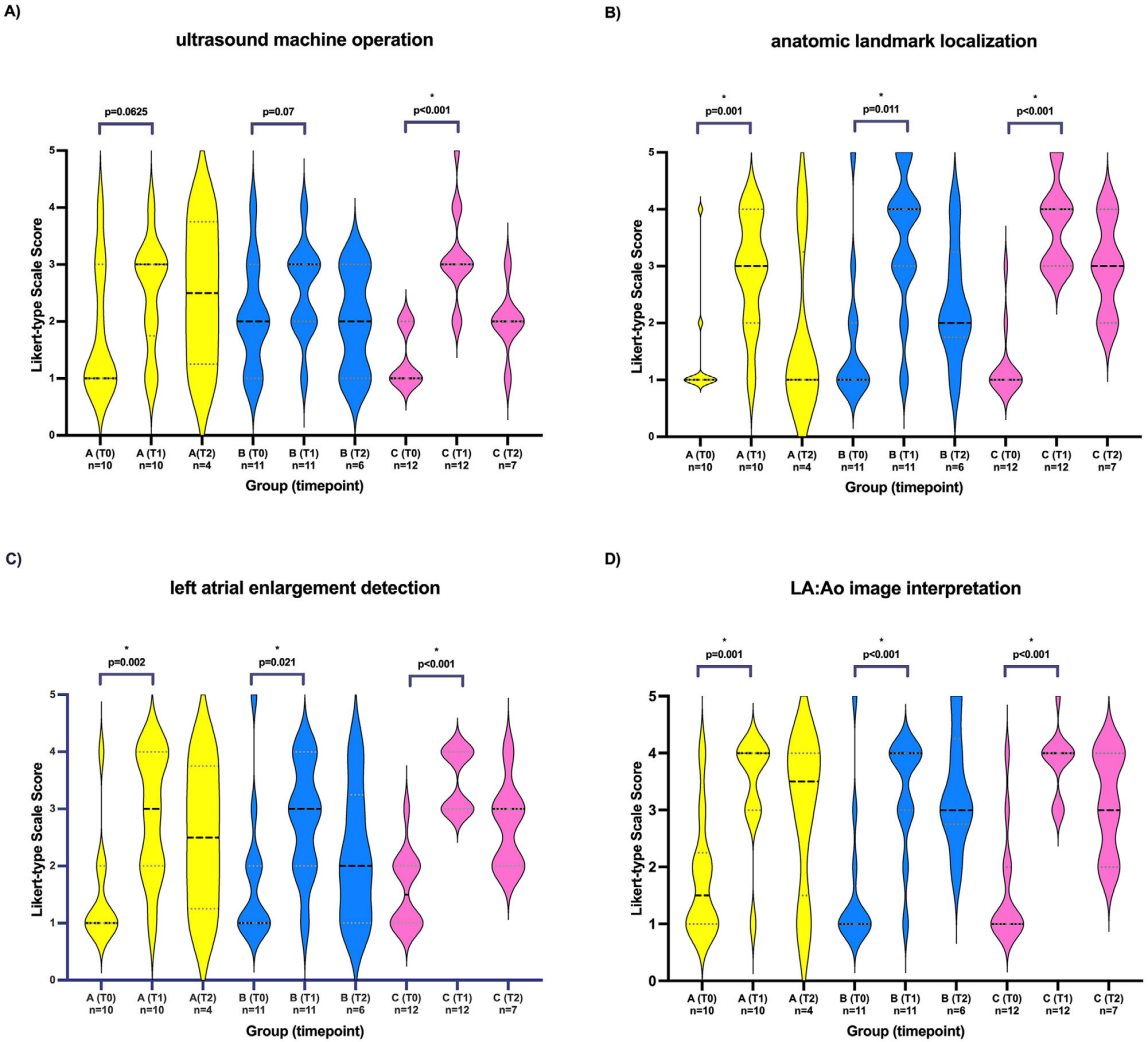
Violin plots showing Likert scale confidence scores obtained for students in Groups A, B, and C regarding (A) ultrasound machine operation, (B) anatomic landmark localization, (C) left atrial enlargement detection, and (D) correct LA:Ao image interpretation before training (T0), immediately after training (T1), and 7–8 months later (T2). All students (Groups A, B, and C) watched a teaching video on acquiring and interpreting the LA:Ao view; Groups B and C then also received in‐person teaching; and Group C additionally received instructor‐led, hands‐on training. Intermittent lines and dots indicate median and IQR, respectively. The asterisk (*) indicates statistically significant difference between T0 and T1 confidence scores with the corresponding *p*‐value. IQR, interquartile range; LA:Ao, left atrial‐to‐aortic ratio.

Likert‐type scale confidence scores obtained for students in Groups A, B, and C regarding ultrasound machine operation, anatomic landmark localization, left atrial enlargement detection, and correct LA:Ao image interpretation over time (T0, T1, and T2) are displayed in Figure 5.

At T0, the median confidence scores in ultrasound machine operation were 1 (IQR: 1–3), 2 (IQR: 1–3), and 1 (IQR: 1–2) in Groups A, B, and C, respectively, with no significant difference detected between the groups (*p* = 0.125). At T1, only Group C showed a significant improvement in median confidence score in ultrasound machine operation (*p* < 0.001). All teaching groups showed improvement in median confidence score in finding anatomic landmarks for the LA:Ao ratio (Group A: *p* = 0.001; Group B: *p* = 0.011; Group C: *p* < 0.001), detecting left atrial enlargement (Group A: *p* = 0.002; Group B: *p* = 0.021; Group C: *p* < 0.001), and interpreting LA:Ao correctly (Group A: *p* = 0.001; Group B: *p* < 0.001; Group C: *p* < 0.001). No difference was found in overall median student confidence scores obtained at T1 between students who successfully acquired an interpretable LA:Ao image (14 [IQR: 11–15.5]) and students who did not (13 [IQR: 12–15]) (*p* = 0.896).

### 3.4 Teaching Method Preferences

At T0, most students enjoyed hands‐on training, with a median Likert‐type scale score of 5 (IQR: 5), and in‐person, lecture‐based teaching, with a median score of 5 (IQR: 4–5). Web‐based teaching was the least favored teaching method, with a median score of 3 (IQR: 2–4). These findings remained true at T1 and T2, with hands‐on training being the most enjoyed teaching method, achieving a median score of 5 (IQR: 5) in all groups. This was followed by in‐person, lecture‐based teaching, with a median score of 5 (IQR: 4–5). Web‐based teaching remained the least favorite teaching method, with a median score of 3 (IQR: 2–4) at both T1 and T2. No difference in the preference of teaching methods between groups was seen at T0 or T1, whether it was web‐based teaching (T0: *p* = 0.473; T1: *p* = 0.430), in‐person teaching (T0: *p* = 0.647; T1: *p* = 0.682), or hands‐on training (T0 + T1: *p* = 0.367). No changes in teaching preferences were observed within any group for any teaching method between T0 and T1. The median Likert‐type scale scores and IQRs of assessed teaching method aspects assessed at T1 are presented in Table 1.

**TABLE 1:** Median Likert scale scores (IQR) immediately following training for survey statements evaluating aspects of teaching method by preclinical veterinary students who watched a teaching video on acquiring and interpreting the LA:Ao view (Groups A, B, and C); who then also received in‐person teaching (Groups B and C); and who then also received instructor‐led, hands‐on training (Group C).

Survey statement	Group A	Group B	Group C	*p*‐value
I enjoy web‐based teaching	4 (2–4)	3 (2–4)	3 (2–4)	0.430
I enjoy in‐person teaching	4.5 (4–5)	5 (4–5)	5 (4–5)	0.682
I enjoy hands‐on practical training	5 (5)	5 (5)	5 (5)	0.367
I enjoyed the teaching course	4 (3–4)	5 (4–5)	5 (5)^a^	0.003
I found the teaching course useful	4 (3–5)	5 (4–5)	5 (5)^a^, ^b^	0.002
I think the teaching course added to my skills as an upcoming veterinarian	4.5 (3–5)	4 (4)	5 (5)^a^, ^b^	0.005
I will try to incorporate the acquired skills and knowledge in future work placements	5 (4–5)	5 (4–5)	5 (4–5)	0.994
I would like to learn more about the sonographic assessment of the heart	5 (5)	5 (5)	5 (5)	0.347
I will recommend this course to my peers	3.5 (2–4)	4 (4–5)	5 (5)^a^, ^b^	<0.001

Abbreviation: IQR, interquartile range.

^a^ Value is significantly higher than in group A (*p* < 0.05).

^b^ Value is significantly higher than in group B (*p* < 0.05).

When the responses to the open‐ended question regarding suggestions for improving the teaching course were analyzed, the most frequent recommendation was for more hands‐on training, cited by 15 of 33 participants. The key themes expressed included enhancing confidence, retention, and understanding through increased practical experience, as reflected in responses such as “Add more hands‐on training to improve confidence, retention, and understanding” and “Opportunity to practice on multiple occasions on other days to cement long‐term knowledge.” Additionally, five of 33 participants suggested that more teaching time would be beneficial, with responses including “Emphasis on the hand movements used with the probe” and “More time spent teaching.” Furthermore, four of 33 participants expressed a preference for a wider variety of dog body conformations, as seen in statements such as “I think maybe a wider range of dogs with different BCS [*body condition scores*] and shapes” and “Have a variety of dog breeds available for looking at the heart.” A comprehensive summary of these responses is provided in Table 2.

**TABLE 2:** Themes emerging from an open‐ended question asking for suggestions for course improvement included in the posttraining survey, provided by preclinical veterinary students who watched a teaching video on acquiring and interpreting the LA:Ao view (Groups A, B, and C); who then also received in‐person teaching (Groups B and C); and who then also received instructor‐led, hands‐on training (Group C).

	Group A (*n* = 10)	Group B (*n* = 11)	Group C (*n* = 12)
More hands‐on training	5	8	2
Longer in‐person demonstration time	2	2	1
Varying body confirmations of dogs and higher numbers of live dogs	0	0	4
Teaching of more advanced cardiac ultrasound skills	3	0	0
Teaching of cardiac abnormalities	0	0	2
More clinical applications	0	1	0
Real cases	1	0	0
In‐person teaching	1	0	0
Operating an ultrasound machine	0	0	1
More example images	1	0	0
More teaching time	0	0	1

*Note*: The yellow, blue, and pink boxes highlight the most often stated suggestion in Groups A, B, and C, respectively. [Color table can be viewed at wileyonlinelibrary.com]

## 4 Discussion

In the current study, there was no difference in successful LA:Ao image acquisition frequency among the groups exposed to the three different teaching methods. This contradicts studies and reviews in human FCU training that conclude that instructor‐led, hands‐on training is important for image acquisition [3, 4, 14]. However, the small sample size may have resulted in a type II error. Although no difference was found among the three groups in the ability to acquire an interpretable LA:Ao image, a greater proportion of students in the instructor‐led, hands‐on training group (Group C) successfully acquired an interpretable image compared with the other groups. This may suggest that hands‐on training and instructor‐led feedback provided additional opportunities for retrieval practice, an act of recalling previously learned information that potentially leads to improved outcomes. However, it is worth noting that the overall proportion of students who were able to acquire an interpretable LA:Ao image was still low at both time points (T1: 13/34; T2: 9/18). International guidelines for teaching FCU in human medicine strongly recommend integrating hands‐on training through supervised ultrasonographic studies, and it likely constitutes an important part of veterinary FCU training as well [3]. Simulation training may be a promising alternative to hands‐on training in live animals because it enables repetitive goal‐directed practice in a controlled, low‐stakes environment. A novel ultrasound training model that uses silicone shapes and ballistic gel has been developed and validated for teaching basic ultrasonography skills in veterinary medicine [15]. Developing a simulator for practicing cardiac ultrasound presents unique challenges due to the dynamic nature of the heart and the continuous anatomic changes that occur, such as variations in left atrial size between systole and diastole. Nevertheless, creating such a simulator remains an intriguing prospect, and further studies that explore the role of simulation in FCU training are warranted.

In the current study, web‐based teaching alone and the addition of in‐person teaching were similarly effective in teaching the technique for LA:Ao image acquisition and interpretation, consistent with findings in human medicine [6]. This suggests that in‐person teaching, without hands‐on training, may not offer additional benefits for teaching practical skills and could potentially be replaced by web‐based teaching. Web‐based teaching is appealing because it is cost‐effective, reusable, time saving for instructors, and continuously available to students with repeated viewing possible, allowing them to study at their own schedule without having to keep up with the instructor's pace. We incorporated retrieval practice through multiple‐choice tests in all groups. The similarities in skills acquisition between web‐based and in‐person teaching may be in part due to the retrieval practice. Replacing in‐person teaching with web‐based teaching conserves educational time for hands‐on training and may be considered when designing future courses that teach POCUS. To further explore the potential of web‐based teaching as a replacement for in‐person teaching, more research is required.

Interestingly, relatively more students in Groups A and B were able to obtain an interpretable LA:Ao image at T2 than at T1, whereas the percentage of students in Group C who succeeded in acquiring an interpretable image did not differ between the two time points. Ultrasonography is a psychomotor skill that involves voluntary limb, joint, and muscle movements in response to sensory stimuli. Key skills for ultrasound include hand–eye coordination (visuomotor skill) and the ability to create a 3D mental image of the anatomy being assessed in relation to other structures (visuospatial skill). Adjusting to different situations and learning to self‐correct are important for mastering the skill of ultrasound performance [16]. At the institution where the current study was conducted, POCUS is routinely used for LA:Ao evaluation. Participating students began their first clinical rotations between T1 and T2, during which they likely observed FCU in clinical practice and had opportunities for self‐directed, hands‐on training. Practicing ultrasound may have contributed to improvement in their hand–eye coordination and LA:Ao image acquisition skills. One may infer from this that students less proficient in ultrasonography are still able to improve their skills in a clinical setting through the effect of retrieval practice. Although students with instructor‐led, hands‐on training were still relatively more successful in LA:Ao image acquisition at both T1 and T2, the marked improvement in image acquisition skill over time may demonstrate that self‐directed, hands‐on training can be beneficial to students and may be an additional resource to improve ultrasonography skills, especially if time and resources are limited. Each of the three groups had students who did not perform as well in the image interpretation test at T2 compared with T1. Self‐directed, hands‐on ultrasound training during clinics is often performed on cardiorespiratory‐stable patients, and students may not have been regularly exposed to patients with an enlarged LA:Ao image. This lack of exposure to the ultrasonographic cardiac appearance of patients with an enlarged left atrium could explain why no improvement in ability to interpret images was seen.

Student confidence in most LA:Ao ultrasound skills was successfully increased by all three teaching methods. When solely assessing for confidence in operating an ultrasound machine, students in Group C, the only group with the opportunity to operate an ultrasound probe and practice LA:Ao image acquisition, were the only ones who experienced increased confidence. At T0, Group C was also the group with the lowest median confidence score in ultrasound machine operation, which could have indicated more scope for improvement. Confidence scores were comparatively lower for all groups at T2 compared with T1. Confidence was evaluated immediately after the teaching intervention(s) at T1 and may have been positively affected by the close proximity of training and evaluation; therefore, the lack of previous teaching intervention at T2 may be reflected in these lower scores.

Interestingly, the decrease in students’ confidence did not translate into their proficiency in LA:Ao image acquisition. Relatively more students in Groups A and B achieved successful image acquisition at T2 than at T1. This lack of correlation between confidence level and proficiency at FCU has been previously noticed in both veterinary and human teaching settings [10, 17].

Hands‐on training was the preferred teaching method for all groups at all time points. This is consistent with a previous study that found hands‐on training to be perceived as the most effective method of POCUS teaching [18]. Despite these students being born in the digital era, web‐based teaching was the least popular teaching method across all groups. Given that students have experienced 3 years of teaching in nonclinical skills and likely predominantly web‐based instruction during the COVID‐19 pandemic, their preference for hands‐on training may reflect their eagerness to engage with the practical aspects of being a veterinarian. In accordance with these findings, students in Group A, having only watched the teaching video, were less satisfied with their assigned teaching method and found it less useful compared with Group C. Groups A and B were also less likely to recommend their teaching course to fellow peers. Most students expressed their desire to learn more about cardiac ultrasound, and more hands‐on training was the most frequent suggestion for improvement of the teaching course. All of these students belonged to Groups A and B, which did not have the opportunity to operate an ultrasound probe and practice LA:Ao image acquisition during their teaching intervention. The findings of this study are echoed in other student veterinary POCUS training programs whereby students consistently expressed a desire for hands‐on training with real‐time instructor feedback [10, 13]. Because of the implications for teaching large cohorts of students, further research on the differences between self‐directed, hands‐on training and instructor‐led, hands‐on training is needed to assess for variations in image acquisition, image interpretation, knowledge retention, and student confidence.

The current study has several limitations. No power analysis was performed to determine the optimal number of students or number of questions in the image assessment. The number of participants was guided by previously published studies of similar design in addition to logistic feasibility. Based on the number of students who successfully acquired an LA:Ao view, a post hoc sample size calculation was performed. With a statistical power of 80% and a 95% CI, 22, 59, and 177 participants per group were required to detect a significant difference in successful image acquisition between Groups A and C, Groups B and C, and Groups A and B, respectively. When the outcome of LA:Ao image acquisition for each group was assessed, the inability to detect a difference between groups may result from a type II error. A larger group of study participants and more test questions may have led to different results. This is especially true for T2, as only 18 of 34 students (53%) could be recruited for this part, mainly because students were on various external teaching placements and not on site. We attempted to mitigate this by choosing two different study days in two consecutive months because students were often off site for several weeks in a row, and students’ availability was requested beforehand. However, due to small group sizes at T2, no statement of knowledge retention can be made. Because of the effect of knowledge decay on long‐term knowledge retention, more studies with larger sample sizes are needed to evaluate the long‐term effect of educational interventions in veterinary medicine.

Allocated teaching times were chosen based on practicability and mirrored possible future student curricula for FCU training. The total training time was 11 min for Group A, 41 min for Group B, and 95 min for Group C. Other studies have ranged from 45 min to 6 h, but various machines and multiple scanning views and measurements were used and thus these studies cannot be directly compared with the current study, which focused on LA:Ao assessment. However, our web‐based video was short compared with instructional content from other studies and may have influenced the effectiveness of the teaching method.

Finally, study participants self‐reported the extent of their previous ultrasound training. Fourth‐year students who had not yet been exposed to clinical rotations were recruited for this study to control for preexisting ultrasonographic experience as best as possible, but previous cardiac ultrasound experience was not an exclusion criterion and may have influenced our results.

In conclusion, no superior teaching method for LA:Ao image acquisition and knowledge retention was found in the current study, but this may be due to small group sizes. Instructor‐led, hands‐on training is popular, may improve student confidence, and likely remains an important aspect of FCU training in veterinary practice. Before any recommendation for FCU teaching to veterinary students can be made, future studies with more participants are needed to explore methods of hands‐on training.

## Ethics Statement

The authors confirm that the ethical policies of the Journal, as noted on the Journal's author guidelines page, have been adhered to and the appropriate ethical review committee approval has been received. The study was approved by the Clinical Research Ethical Review Board (URN 2022 2139‐2) and the Social Sciences Research Ethical Review Board (SR2022‐0003) at The Royal Veterinary College, Hatfield, UK.

## Conflicts of Interest

The authors declare no conflicts of interest.

## Supporting information




**Supporting File 1**: vec70104‐sup‐0001‐SuppMat.docx

**Supporting File 2**: vec70104‐sup‐0002‐SuppMat.mov

**Supporting File 3**: vec70104‐sup‐0003‐SuppMat.docx

**Supporting File 4**: vec70104‐sup‐0004‐SuppMat.pdf

**Supporting File 5**: vec70104‐sup‐0005‐SuppMat.docx

## Endnotes

1 Randomizer.org (https://www.randomizer.org), accessed January 28, 2023.

2 Butterfly Network, Inc. Butterfly iQ+ Vet [ultrasound probe]. Burlington, MA: Butterfly Network, Inc; 2021.

3 Apple Inc. iPad Pro (third generation) [tablet computer]. Cupertino, CA: Apple Inc; 2018.

4 Zaiontz C. Real Statistics Using Excel. 2023; http://www.real‐statistics.com, accessed February 2, 2024.
