# Supplementary material for: Assessing the Effect of Different Teaching Methods on Left Atrial‐to‐Aortic Ratio Image Acquisition and Image Interpretation
Source: J Vet Emerg Crit Care (San Antonio). 2026 Apr 15;36(2):158–67. doi: 10.1111/vec.70104 (PMC13150402; doi:10.1111/vec.70104)
Supplement: Supplementary file 3 — Supporting File 3: vec70104‐sup‐0003‐SuppMat.docx [file VEC-36-158-s003.docx]

POST-INTERVENTION SURVEY

1. Please indicate your preference for different teaching methods:

| Strongly disagree | 1 |
| --- | --- |

| Strongly agree | 5 |
| --- | --- |

|  | 1 | 2 | 3 | 4 | 5 |
| --- | --- | --- | --- | --- | --- |
| I enjoy In-person teaching |  |  |  |  |  |
| I enjoy Online teaching |  |  |  |  |  |
| I enjoy Hands-on practice training |  |  |  |  |  |

1. What teaching intervention group were you assigned to?

- Web-based training course only
- Web-based training course + in-person didactic training
- Web-based training course + in-person didactic training + hands-on training

1. Please indicate your opinion on this teaching course:

| Strongly disagree | 1 |
| --- | --- |

| Strongly agree | 5 |
| --- | --- |

|  | 1 | 2 | 3 | 4 | 5 |
| --- | --- | --- | --- | --- | --- |
| I enjoyed the teaching course |  |  |  |  |  |
| I found the teaching course useful |  |  |  |  |  |
| I think the teaching course added to my skills as an upcoming veterinarian |  |  |  |  |  |
| I will try to incorporate the acquired skills and knowledge in my future work placements |  |  |  |  |  |
| I would like to learn more about the assessment of the heart with ultrasound |  |  |  |  |  |

1. Would you take another course on a different topic but similar in design like the one assigned to you?

Yes No

1. Please indicate your opinion on the following statement:

| Strongly disagree | 1 |
| --- | --- |

| Strongly agree | 5 |
| --- | --- |

|  | 1 | 2 | 3 | 4 | 5 |
| --- | --- | --- | --- | --- | --- |
| I will recommend this course to my peers |  |  |  |  |  |

1. In what ways would you improve this course?
2. Please indicate your opinion on the following statements:

| Strongly disagree | 1 |
| --- | --- |

| Strongly agree | 5 |
| --- | --- |

|  | 1 | 2 | 3 | 4 | 5 |
| --- | --- | --- | --- | --- | --- |
| I feel confident in operating an ultrasound machine |  |  |  |  |  |
| I feel confident in using ultrasound to detect left atrial enlargement in dogs |  |  |  |  |  |
| I feel confident in finding the anatomic landmarks to assess an LA:Ao ratio |  |  |  |  |  |
| I feel confident in correctly interpreting an LA:Ao ratio |  |  |  |  |  |
