# Supplementary material for: Assessing the Effect of Different Teaching Methods on Left Atrial‐to‐Aortic Ratio Image Acquisition and Image Interpretation
Source: J Vet Emerg Crit Care (San Antonio). 2026 Apr 15;36(2):158–67. doi: 10.1111/vec.70104 (PMC13150402; doi:10.1111/vec.70104)
Supplement: Supplementary file 4 — Supporting File 4: vec70104‐sup‐0004‐SuppMat.pdf [file VEC-36-158-s004.pdf]

A Case-based Single Choice Question Evaluation

Manual for SCQ

Dear participant!

Focused Cardiac Ultrasound, an ultrasound technique designed for an emergency assessment of the heart, forms part of the human medical curriculum but is not currently part of the veterinary curriculum. Various methods of training have been described in human medicine, but it is unclear how the difference methods affect skill acquisition, knowledge acquisition and confidence in FCU. The aim of our study is to compare these outcomes between three different methods of teaching FCU.

You are about to sit the first part of the study – our short Single-Choice Question (SCQ) test:

In this test we will ask you to interpret five different ultrasound images obtained from a dog, for the assessment of the left atrial-to-aortic ratio (LA:Ao). The questions will be presented based on assumptive cases presenting through the Emergency Service. However, in this SCQ we solely want you to interpret the sonographic image, not taking into account the history or clinical examination of the dog presented.

The three possible answers to the SCQ are: normal LA:Ao, enlarged LA:Ao or not interpretable view.

This SCQ is a single choice only test, therefore only one answer will be the correct answer and we would like you to select only one answer out of these three.

You will be asked to answer the questions step by step. Once an answer to a question has been selected and you chose to move on to the next image, your answer will be logged and you will not be able to revisit the question.

Case 1

A 5 year old male entire Miniature Dachshund presents to your Emergency Service with a history of progressive exercise intolerance and new onset tachypnea. Clinical examination reveals a tachypnea of 44 breaths per minute but is otherwise unremarkable. You perform a focused cardiac ultrasound for an emergency assessment of the heart and obtain the following image. How do you interpret this image in light of LA:Ao assessment?

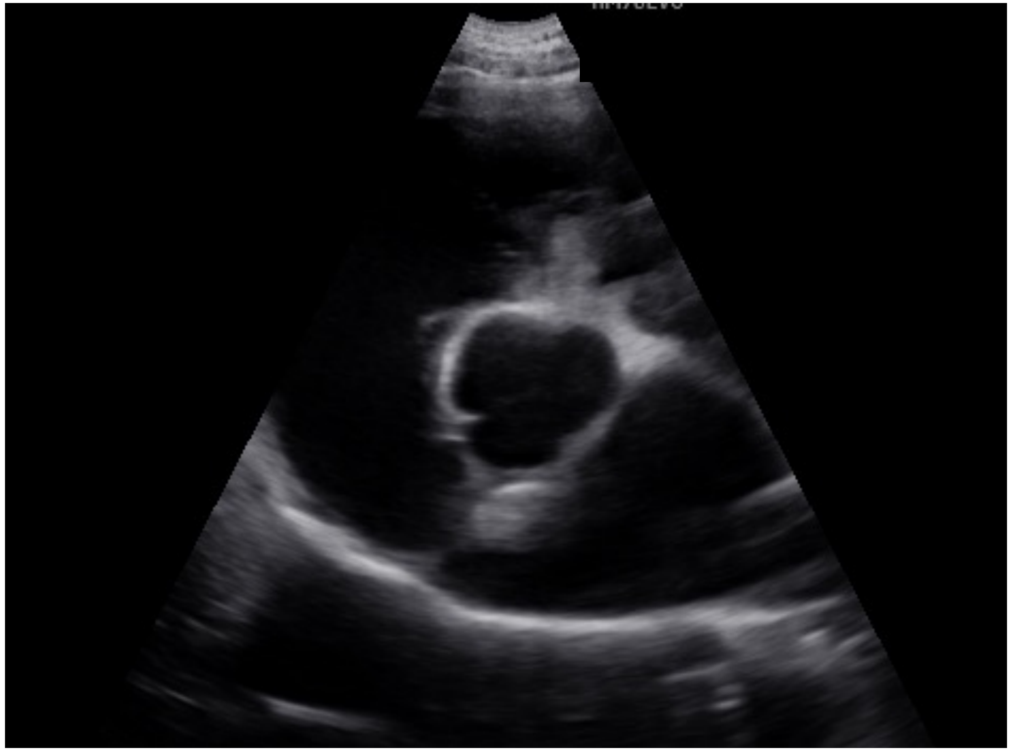

- ☐ LA:Ao enlarged
- ☐ LA:Ao within normal limits
- ☐ Not interpretable view of LA:Ao

Case 2

A 10 year old female entire Labrador Retriever presents to your Emergency Service with a history of progressive lethargy and trouble breathing. Clinical examination reveals an arrythmia with weakened pulse quality and a tachypnea of 40 breaths per minute. You perform a focused cardiac ultrasound for an emergency assessment of the heart and obtain the following image. How do you interpret this image in light of LA:Ao assessment?

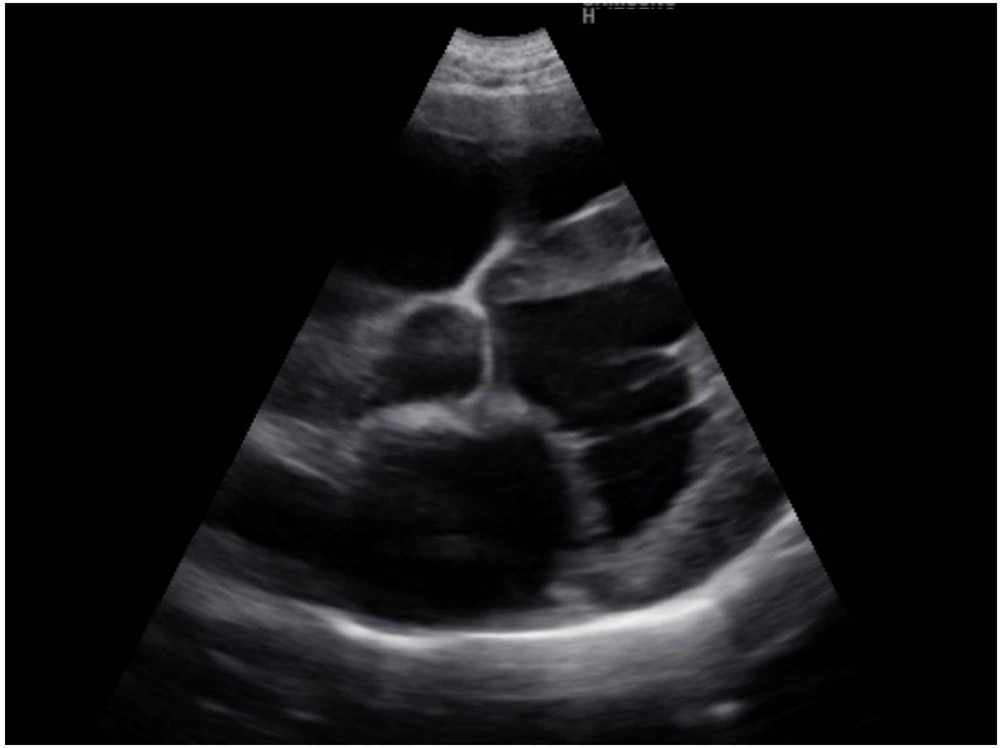

☐ LA:Ao enlarged
☐ LA:Ao within normal limits
☐ Not interpretable view of LA:Ao

Case 3

An 8 year old male neutered West Highland White Terrier presents to your Emergency Service with a 1-month history of a cough, and progressive dyspnea. Clinical examination reveals a tachypnea of 60 breaths per minute with increased abdominal effort but is otherwise unremarkable. You perform a focused cardiac ultrasound for an emergency assessment of the heart and obtain the following image. How do you interpret this image in light of LA:Ao assessment?

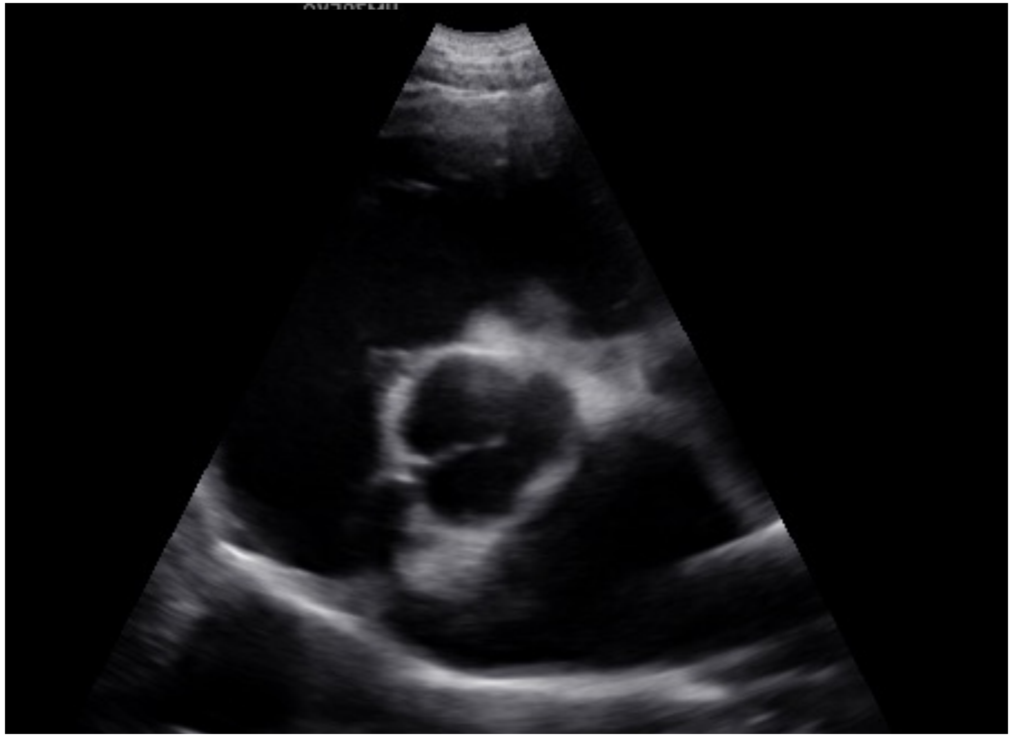

☐ LA:Ao enlarged
☐ LA:Ao within normal limits
☐ Not interpretable view of LA:Ao

Case 4

A 12 year old Chihuahua presents to your Emergency Service with a history of new onset dyspnea, which progressively worsened over the last 24 hours. Clinical examination reveals a tachypnea of 66 breaths per minute with increased abdominal effort. You perform a focused cardiac ultrasound for an emergency assessment of the heart and obtain the following image. How do you interpret this image in light of LA:Ao assessment?

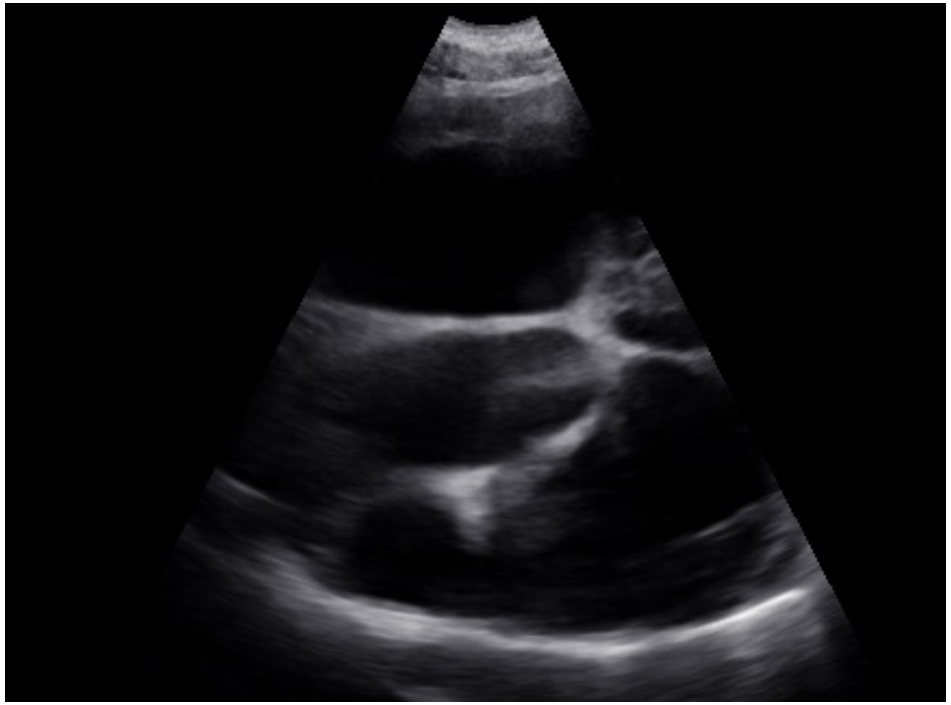

- ☐ LA:Ao enlarged
- ☐ LA:Ao within normal limits
- ☐ Not interpretable view of LA:Ao

Case 5

A 7 year old male neutered Cocker Spaniel presents to your Emergency Service with a history of progressive exercise intolerance and new onset tachypnea. Clinical examination reveals a tachypnea of 56 breaths per minute with increased effort. You perform a focused cardiac ultrasound for an emergency assessment of the heart and obtain the following image. How do you interpret this image in light of LA:Ao assessment?

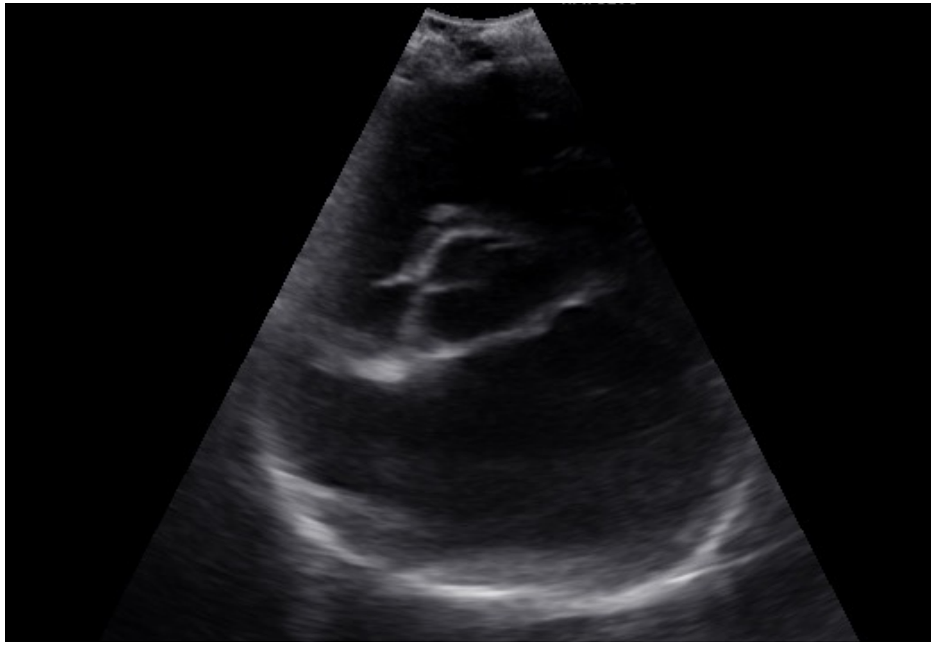

- ☐ LA:Ao enlarged
- ☐ LA:Ao within normal limits
- ☐ Not interpretable view of LA:Ao
