# Supplementary material for: Assessing the Effect of Different Teaching Methods on Left Atrial‐to‐Aortic Ratio Image Acquisition and Image Interpretation
Source: J Vet Emerg Crit Care (San Antonio). 2026 Apr 15;36(2):158–67. doi: 10.1111/vec.70104 (PMC13150402; doi:10.1111/vec.70104)
Supplement: Supplementary file 5 — Supporting File 5: vec70104‐sup‐0005‐SuppMat.docx [file VEC-36-158-s005.docx]

PRE-EVALUATION SURVEY AT T2

1. Please indicate your preference for different teaching methods:

| Strongly disagree | 1 |
| --- | --- |

| Strongly agree | 5 |
| --- | --- |

|  | 1 | 2 | 3 | 4 | 5 |
| --- | --- | --- | --- | --- | --- |
| I enjoy In-person teaching |  |  |  |  |  |
| I enjoy Online teaching |  |  |  |  |  |
| I enjoy Hands-on practice training |  |  |  |  |  |

1. Have you operated an ultrasound machine on small animals in the last six months?

Yes No

1. If your answer to the previous question was yes: How many times in total have you operated an ultrasound machine?

- Once
- 2 - 5 times
- 6 – 10 times
- > 10 times

1. How many times have you used ultrasound to assess cardiac structure and/or function in the last six months?

- Once
- 2 - 5 times
- 6 – 10 times
- > 10 times

1. Please indicate your opinion on each of the following statements:

| Strongly disagree | 1 |
| --- | --- |

| Strongly agree | 5 |
| --- | --- |

|  | 1 | 2 | 3 | 4 | 5 |
| --- | --- | --- | --- | --- | --- |
| I feel confident in operating an ultrasound machine |  |  |  |  |  |
| I feel confident in using ultrasound to detect left atrial enlargement in dogs |  |  |  |  |  |
| I feel confident in finding the anatomic landmarks for assessing an LA:Ao ratio |  |  |  |  |  |
| I feel confident in correctly interpreting an LA:Ao ratio |  |  |  |  |  |
